# Effect of Copper Cobalt Oxide Composition on Oxygen Evolution Electrocatalysts for Anion Exchange Membrane Water Electrolysis

**DOI:** 10.3389/fchem.2020.600908

**Published:** 2020-11-04

**Authors:** Chae-Yeon Kwon, Jae-Yeop Jeong, Juchan Yang, Yoo Sei Park, Jaehoon Jeong, Honghyun Park, Yangdo Kim, Sung Mook Choi

**Affiliations:** ^1^Materials Center for Energy Convergence, Surface Technology Division, Korea Institute of Materials Science (KIMS), Changwon, South Korea; ^2^School of Materials Science and Engineering, Gyeongsang National University, Jinju, South Korea; ^3^Department of Materials Science and Engineering, Pusan National University, Busan, South Korea; ^4^Department of Advanced Biomaterials Research, Materials Processing Innovation Research Division, Korea Institute of Materials Science (KIMS), Changwon, South Korea

**Keywords:** oxygen evolution reaction (OER), coprecipitaion method, electrolysis, anion exchange membrane (AEM), hydrogen production

## Abstract

Copper cobalt oxide nanoparticles (CCO NPs) were synthesized as an oxygen evolution electrocatalyst *via* a simple co-precipitation method, with the composition being controlled by altering the precursor ratio to 1:1, 1:2, and 1:3 (Cu:Co) to investigate the effects of composition changes. The effect of the ratio of Cu^2+^/Co^3+^ and the degree of oxidation during the co-precipitation and annealing steps on the crystal structure, morphology, and electrocatalytic properties of the produced CCO NPs were studied. The CCO_1:2_ electrode exhibited an outstanding performance and high stability owing to the suitable electrochemical kinetics, which was provided by the presence of sufficient Co^3+^ as active sites for oxygen evolution and the uniform sizes of the NPs in the half cell. Furthermore, single cell tests were performed to confirm the possibility of using the synthesized electrocatalyst in a practical water splitting system. The CCO_1:2_ electrocatalyst was used as an anode to develop an anion exchange membrane water electrolyzer (AEMWE) cell. The full cell showed stable hydrogen production for 100 h with an energetic efficiency of >71%. In addition, it was possible to mass produce the uniform, highly active electrocatalyst for such applications through the co-precipitation method.

## Introduction

Due to climate change caused by the use of fossil fuels and the acceleration of industrialization, a shift in the global energy paradigm is necessary. Of the options available, hydrogen is a notable resource that could replace existing fossil fuels due to its high energy density, environmental credentials, and practically infinite sources (Kang et al., 2007; Khan et al., 2018). Hydrogen production is one of the most important aspects that must be considered for the development of a hydrogen energy-based society. Currently, hydrogen production methods using natural gas or by-product hydrogen (gray hydrogen) (Turner, 2004) are widely employed. However, these methods utilize existing fossil fuels or are associated with additional environmental pollution factors. Therefore, the water electrolysis method, which produces hydrogen from water as a perfectly eco-friendly form of energy and does not use fossil fuels or release CO_2_, is drawing attention (Chu and Majumdar, 2012; Xu et al., 2016). Major methods of water electrolysis are alkaline water electrolysis (AWE), which is based on liquid electrolytes, and proton exchange membrane water electrolysis (PEMWE), which is based on solid electrolytes. The former is a commercialized system that can use a non-precious catalyst and is cost effective due to its operation in an alkaline atmosphere. However, operations based on liquid electrolytes have drawbacks such as low efficiency, corrosion due to the high concentration of KOH, difficulties when undertaking high pressure operations, and difficult facility maintenance. On the other hand, solid electrolyte-based systems enable high pressure charging, the production of high purity hydrogen, as well as high efficiency and stability. However, PEMWE also has a great disadvantage in that it requires the use of expensive precious metal catalysts due to the acidic atmosphere (Muradov and Veziroglu, 2005; Balat, 2007; Sobrino et al., 2010).

A next generation electrolysis system that was developed to secure the advantages of both these systems is anion exchange membrane water electrolysis (AEMWE). AEMWE can use inexpensive non-precious catalysts for operation in alkaline solutions such as KOH, yields high purity hydrogen *via* the use of a solid electrolyte membrane, and can operate at high pressures, which can reduce the unit cost of production and increase efficiency. However, its technological intensity to date is less than those of the other two systems, and it has the task of improving both the durability of the AEM and the performance of the non-precious metal catalysts. In particular, there is an urgent need to develop a highly active electrocatalyst as the oxygen evolution reaction (OER) at the anode becomes the rate-limiting step of the entire system.

Presently, the most active OER electrocatalysts are Ru- and Ir-based oxides. The high cost of these precious metal catalysts is a major stumbling block to commercial applications that require mass production, and there are ongoing studies into non-precious electrochemical catalysts to replace them (Bagheri et al., 2016; Choi et al., 2018). Among the non-precious metal electrocatalysts, transition metal oxides of Ni, Co, and Fe are reported to show high oxygen evolution performances (Wu and Scott, 2011; Bikkarolla and Papakonstantinou, 2015; Liu et al., 2016; Cheng et al., 2017; Aqueel Ahmed et al., 2018; Xu et al., 2018; Wang et al., 2019), with copper-cobalt oxide (CCO) electrocatalysts being composed of low-priced transition metals and having high stabilities due to reverse spinel structures of Co and high conductivity *via* partial replacement of Cu. Various methods have been used to synthesize CCO OER electrocatalysts, including chemical deposition, hydrothermal methods, electrodeposition, and coprecipitation; however, it is most important that uniform electrocatalysts are obtained in sufficiently large quantities to be applied to the system. We synthesized CCO through a coprecipitation method that allows for simple mass production with highly active non-precious metal electrocatalysts. The physiochemical and structural electrochemical properties of these catalysts were studied as the ratio of precursors used for the coprecipitation was changed, and the applicability of the actual system was confirmed by both developing the electrocatalysts and applying it to water electrolysis single cells.

## Experimental Section

### Synthesis of CCO OER Electrocatalyst

CCO electrocatalysts with different ratios of precursors were synthesized using CuSO_4_·5H_2_O (Sigma Aldrich) and Co(NO_3_)_2_·6H_2_O (Sigma Aldrich) in 1:1, 1:2, and 1:3 (Cu:Co) molar ratios. The concentration of Co was fixed at 100 mM. Each Cu and Co precursor was dissolved in 4 L of distilled water (Milli-Q, Merck, 18.2 MΩ·cm) for 30 min, and then ammonia solution (NH_4_OH, 28–30%, SAMCHUN) was used for coprecipitation. The pH of the solution was fixed at 9.5 and the ammonia solution was injected at a speed of 5 ml/min. After coprecipitation, the precursor solutions were stirred for 3 h and aged for 1 h to allow for stabilization. After the stabilization process was completed, the precipitate was separated from the solution *via* centrifuge (HERMLE, X36HK), quickly cooled using liquid nitrogen, and freeze-dried for 24 h at −95°C. After drying, the electrocatalyst was pulverized using a mortar, and heat treatment was carried out for 4 h at 300°C at a heating rate of 10°C/min in an air atmosphere. After 2 h of heat treatment, the particles were evenly crushed *via* ball milling (SPEX, 8000D) to yield the CCO. All processes except the precursor ratios were consistent between samples, and the catalysts with different Cu:Co ratios were named CCO_1:1_, CCO_1:2_, and CCO_1:3_, respectively. The overall catalytic synthesis is shown in Supplementary Figure 1.

### Physical Characterization

The microstructures of CCOs synthesized with different precursor ratios were identified using field emission scanning electron microscopy (FE-SEM, JEOL, JSM-7001F) and transmission electron microscopy (TEM, JEOL, JEM 2100F) for size and morphology, while X-ray diffraction (XRD) analysis was used to analyze their crystallinities. XRD patterns were recorded using a 2θ scan from 20 to 80° with Cu-Kα radiation of 1.540 Å (40 kV, 300 mA). In addition, atomic compositions and bindings of CCO surfaces were analyzed through X-ray photoelectron spectroscopy (XPS, ThermoFisher, K-alpha). Inductively coupled plasma (ICP) emission spectrometer (Agilent, Agilent 5110) analysis was used to determine the content of various elements. The specific surface areas and pore structures of the synthesized electrocatalysts were measured using a MicrotracBEL (BELSORP-MAX), and were calculated using the nitrogen adsorption isothermal lines (N_2_/77 K). S_BET_ (specific surface area) was estimated using the Brunauer-Emmett-Teller (BET) formula, and the total pore volume (V_t_) was obtained through the amount of nitrogen adsorbed at P/P_0_ = 0.99. In addition, the mesopore volume was obtained using the Barrett-Joyner-Halenda (BJH) equation, and the average porous diameter (D) was obtained using the following Equation(1):

(1) $$\begin{array}{l} \text{D}\left(\text{nm}\right)=\frac{{4V}_{t}\;\times 1000}{S_{BET}} \end{array}$$

### Electrochemical Characterization

Linear sweep voltammetry (LSV) was carried out using a potentiostat (BioLogic, VMP3) for the electrochemical characterization of the CCOs. The three-electrode system used a graphite rod and Hg/HgO (1 M KOH) as a counter electrode and reference electrode, respectively, for experiments in alkaline media. A KOH solution (1 M) was used as the electrolyte. Rotating disk electrodes (RDE, ALS, glassy carbon) with an area of 0.07 cm^2^ were used as working electrodes. The RDE was polished consecutively with two different alumina powders (0.3 and 0.05 μm) and cleaned by sonication for 20 min in deionized water to remove the alumina powders from the electrodes. The electrocatalyst power (20 mg) was added to 850 μl of ethanol and 150 μl of 5 wt.% Nafion (D521 Nafion™ Dispersion), and upon sonication for 15 min, an ink containing the uniformly distributed electrocatalyst was produced. A droplet of the electrocatalyst ink (3 μl) placed on the RDE was dried at 80°C for 1 min. All potentials were reported vs. a reversible hydrogen electrode (RHE) (V_RHE_ = V vs. RHE).

### AEMWE Single Cell Characterization

To evaluate the performances of the synthesized electrocatalysts in the water electrolysis system, electrodes were formulated and applied to the system. The synthesized CCO (1 g) was mixed with distilled water (0.8 g), isopropanol (0.4 g, 99.5%), and 3 wt.% Nafion (0.6 g) as a binder to produce a slurry. Anodes and cathodes were prepared by the catalyst coated substrate (CCS) method. The slurry was hot-pressurized at a pressure of 100 kg/cm^2^ at 140°C on Ni foam (NF, Alantum, pore size: 450 μm) to produce an anode. In addition, Pt/C (40 wt.%, HISPEC 4,000, Johnson Matthey) was placed on carbon cloth in a loading amount of 1 mg_Pt_/cm^−2^ to produce a cathode. AEMWE single cells were also tested with an anion exchange membrane (AEM, X37-50 Grade T, Dioxide Materials). The single cell formed was circulated at a temperature of 60°C and a flow rate of 50 mL min^−1^, and the cyclic voltammetry (CV) method was employed at a rate of 10 mV s^−1^ within a potential range of 1.6–1.9 V_cell_ (voltage in system) for cell activation. After stabilizing the cell, electrochemical analysis was performed at a fixed cell temperature of 50°C.

## Results and Discussion

**Figure 1:**
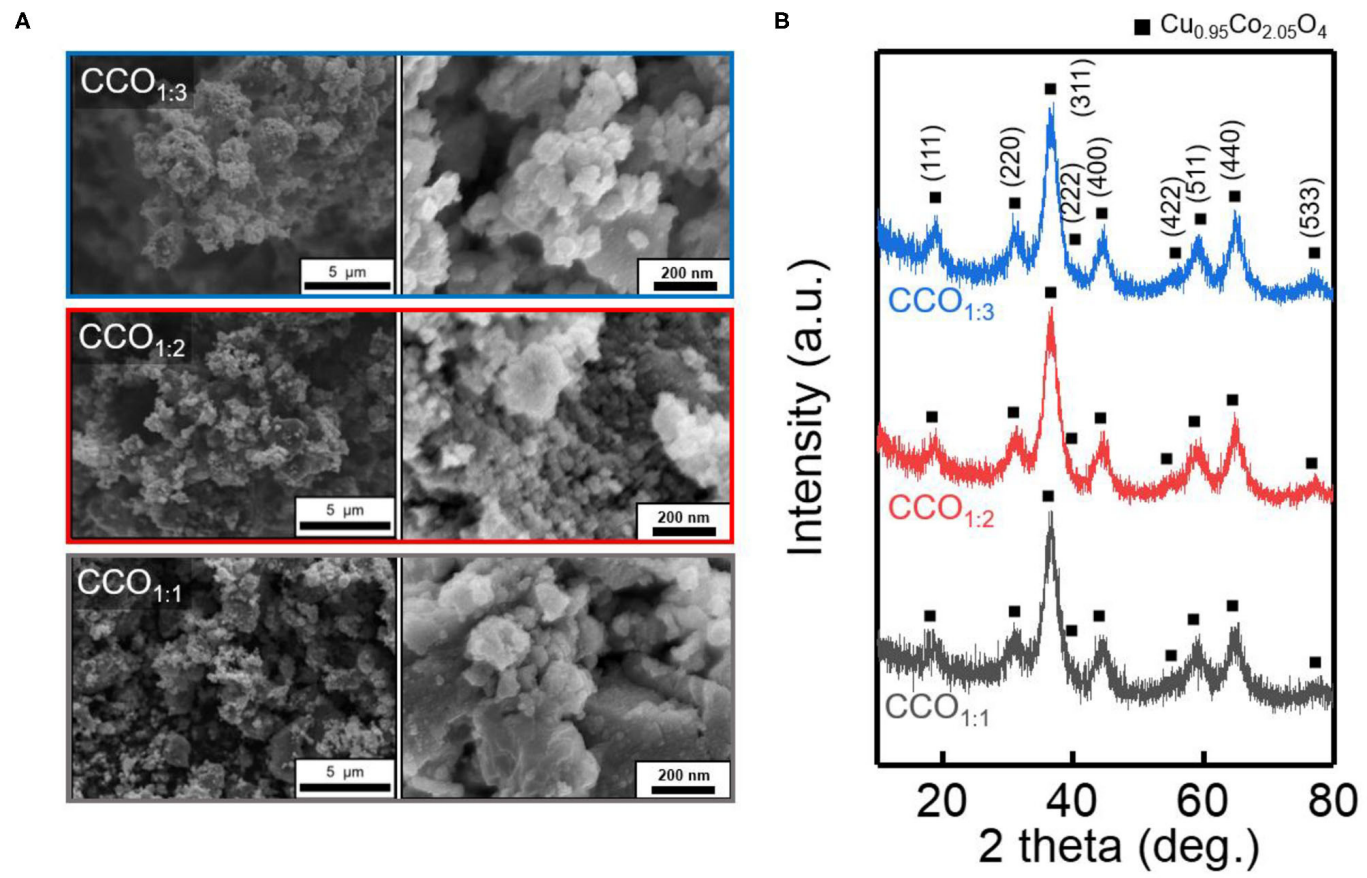
Morphology and crystal structure of synthesized CCO electrocatalyst. **(A)** Scanning electron microscopic (SEM) image and **(B)** X-ray diffraction (XRD) pattern with the different precursor ratio. XRD confirms the formation of the Cu_0.95_Co_2.05_O_4_ (ICSD:98-006-3387).

Figure 1A shows SEM images demonstrating the changes in the morphology of the CCO electrocatalyst as the ratio of Cu to Co was altered. The pH was increased by the addition of NH_4_OH to the precursor solution, leading to changes in the solubilities of the ions and thus deposition of Cu and Co ions (Jang et al., 2020). When the sediment was finally obtained through the oxidation heat treatment process, all the final electrocatalysts were found to consist of uniform nanoparticles that were agglomerated together to form secondary particles. Figure 1B shows the XRD data used to identify the structures and crystallinities of the electrocatalysts. The synthesized electrocatalysts all consisted of Cu_0.95_Co_2.05_O_4_ (ICSD No. 98-006-3387) in a reverse-spinel structure, which is known to have excellent OER performance (Park S. M. et al., 2020). For all electrocatalysts that were synthesized with various precursor ratios, the main (311) peak was observed at 36.62°, and the same 2θ values were identified for planes (111), (220), (222), (422), (540), and (533) (Chi et al., 2008). Furthermore, the lattice parameters and average particle sizes of the strong (311) peak were identified using Bragg's Equation (2) and the Debye-Scherrer Equation (3). In these formulae, K is a dimensionless shape factor, λ is the X-ray wavelength (Cu Kα = 1.54 Å), θ is the Bragg angle, and β_1/2_ is the full width at half maximum of the peak (Marsan et al., 1992; Mendonca et al., 2002).

(2) $$\begin{array}{l} \text{a}=\frac{\lambda }{2\sin\theta }\times \sqrt{h^{2}+k^{2}+1^{2}} \end{array}$$

(3) $$\begin{array}{l} \text{d}=\frac{K\lambda }{\beta_{1/2}\cos\theta } \end{array}$$

The calculated lattice constant of the (311) plane was a = 8.12 Å, which is similar with a previous study (a = 8.10 Å) (Gautier et al., 1997). The average particle sizes of CCO_1:1_, CCO_1:2_, and CCO_1:3_ were 3.09, 2.82, and 3.2 nm, respectively. As mentioned earlier, all CCOs had reverse spinel structures (Jang et al., 2020)and were in the Fd3m space group, with Co^3+^ occupying the tetrahedral positions and Cu^2+^ and Co^3+^ occupying the octahedral positions (Gautier et al., 1997).

**Figure 2:**
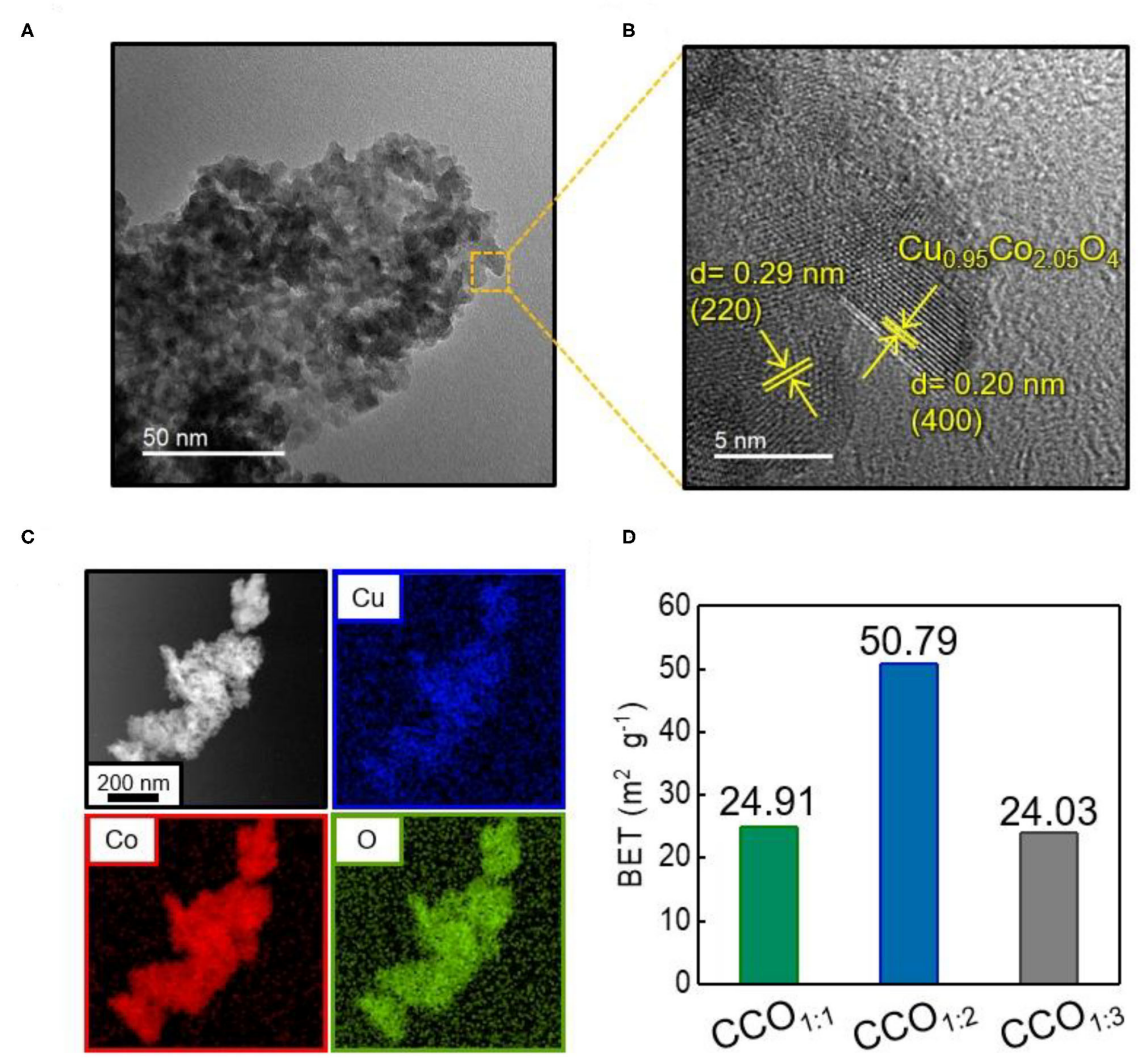
Identification of CCO_1:2_ electrocatalyst **(A)** Transmission electron microscopic (TEM) image of CCO_1:2_. **(B)** High-resolution TEM image of magnifying the area indicated by the square in **(A)**. **(C)** Energy dispersive spectrometry (EDS) elemental mapping of Cu (blue), Co (red), and O (green) in CCO_1:2_, showing a homogeneous dispersion of all elements. **(D)** The comparison of the surface area of CCO_1:1_, CCO_1:2_, CCO_1:3_. Surface areas were calculated by using the Brunauer, Emmet, and Teller (BET) model: 24.91 m^2^·g^−1^ for CCO_1:1_, 50.79 m^2^·g^−1^ for CCO_1:2_ and 24.03 m^2^·g^−1^ for CCO_1:3_.

There is little difference between the diffraction angles of the (hkl) indices of CuCo_2_O_4_ and Co_3_O_4_, making it difficult to distinguish between the two by XRD analysis alone (Song et al., 2020). Thus, the shape and microstructure of CCO_1:2_ were analyzed *via* TEM (Figure 2). Uniform spherical nanoparticles with sizes of 3–5 nm were observed in line with a prior study in which ammonia solution (NH_4_OH) was adjusted to pH 9.5, and these nanoparticles were concentrated together into one mass (Figure 2A) (Park S. M. et al., 2020). Further analysis of a high resolution image of the square indicated in Figure 2A led to d-spacing values of 0.29 and 0.20 nm, indicating (220) and (400) planes, respectively, which was in agreement with the XRD pattern results for Cu_0.95_Co_2.05_O_4_ (Figure 2B) (Silambarasan et al., 2016). The constituent elements of CCO_1:2_ were identified as Cu, Co, and O by energy-dispersive X-ray spectroscopy (EDS, Figure 2C). The overall uniform distribution plot confirmed that these elements were evenly dispersed throughout the CCO nanoparticles. In addition, the change in the surface area of the electrocatalysts as the Cu:Co ratio was adjusted was measured *via* BJH analysis (Figure 2D). It was found that CCO_1:2_ had the highest surface area, at 50.79 m^2^/g; this was twice as high as those of CCO_1:1_ and CCO_1:3_ (Supplementary Figure 2). Additionally, the average pore diameter of CCO_1:2_ was 8.7 nm. Since a high specific surface area is a requirement for highly active electrocatalysts, this contributed to the improved catalytic activity of CCO_1:2_, as discussed later.

**Figure 3:**
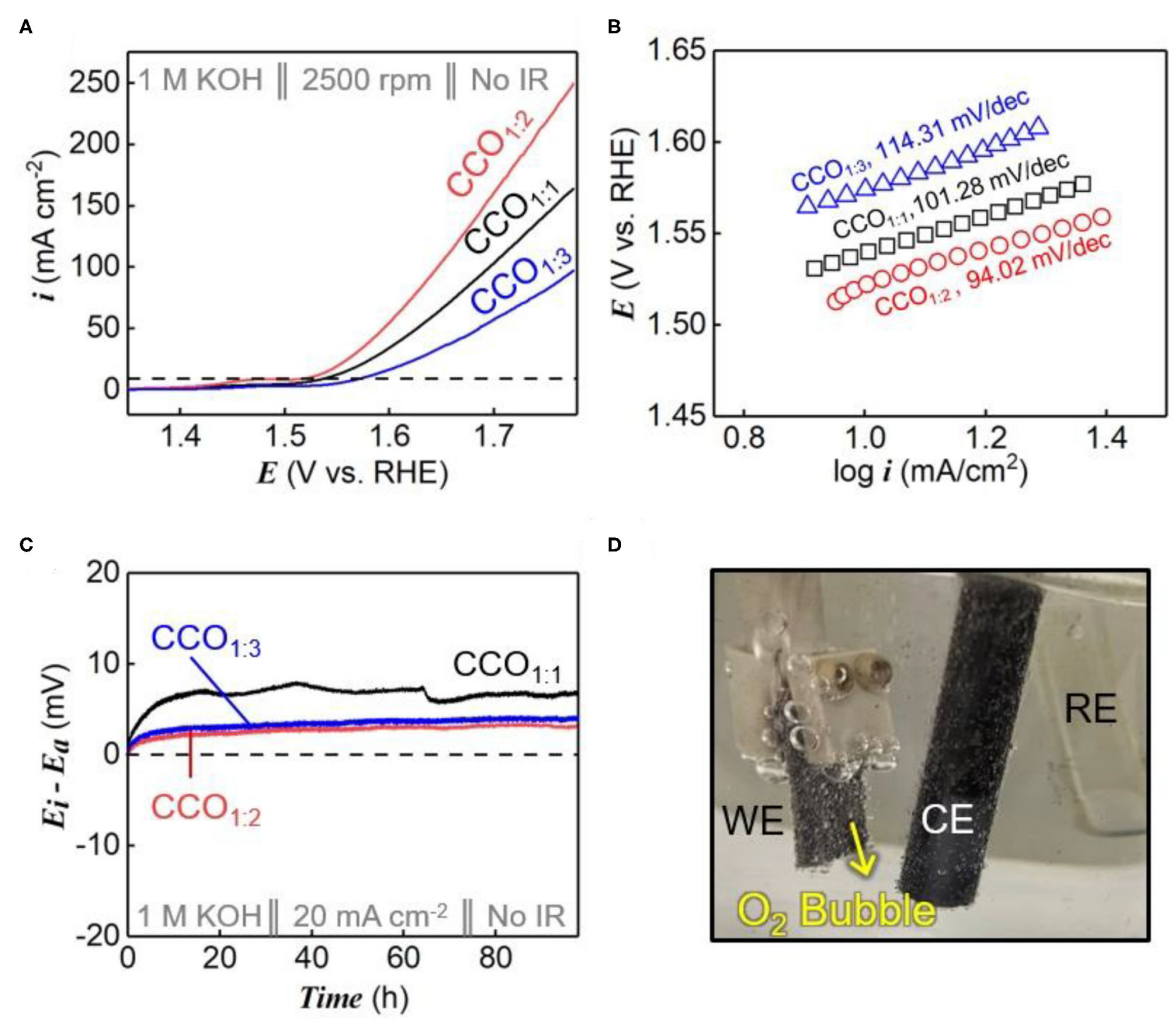
OER performance. **(A)** The LSV polarization curves of CCO_1:1_, CCO_1:2_, and CCO_1:3_ in 1 M KOH at a scan rate of 5 mV s^−1^. **(B)** Tafel plots derived from **(A)**. **(C)** The durability test of CCO_1:1_, CCO_1:2_, and CCO_1:3_ for 100 h at 20 mA cm^−2^. **(D)** The image of continuously generated oxygen bubble during the durability test.

Figure 3 shows the OER performances of CCO electrocatalysts synthesized using different precursor ratios, as determined by electrochemical analysis. To identify the electrocatalytic characteristics of the synthesized catalysts, their electrocatalytic activities were evaluated using RDEs at a scan rate of 5 mV/s in 1 M KOH electrolyte (Figure 3A). The overpotentials (η) at a current density of 10 mA/cm^2^ (dotted line within Figure 3A) were used to compare the relative catalytic activities, and were measured as 310, 292, and 344 mV_RHE_ for CCO_1:1_, CCO_1:2_, and CCO_1:3_, respectively. As CCO_1:2_ had the lowest overpotential value, it was capable of more rapid oxygen evolution than the other electrocatalysts. As shown in Figure 3B, the Tafel slopes were calculated using the Tafel equation, as shown in Equation (4):

(4) $$\eta =\text{b log}\left(\text{i}/{\text{i}}_{0}\right),\text{ where b}=\text{RT}/\alpha \text{NF}$$

where η represents overpotential, b is the Tafel slope, i is the current density, i_0_ is the exchange current density, R is the gas constant (8.314 J/K·mol), α is the symmetry factor (0.5), and F is the Faraday constant (96,485 C mol^−1^). CCO_1:1_, CCO_1:2_, and CCO_1:3_ had Tafel slopes of 101.28, 94.02, and 114.31 mV/dec, respectively. The lower slope of CCO_1:2_ demonstrates an improved response rate over the other electrocatalysts, as a smaller Tafel slope results in a lower overvoltage with increased current density. This in turn shows that CCO_1:2_ had superior catalytic activity over those prepared with other precursor ratios (Pendashteh et al., 2014).

Figure 3C shows the results of durability tests for all the synthesized electrocatalysts. To ensure accurate measurements, a fixed amount of electrocatalyst was placed on the Ni foam to fabricate an electrode with a size of 1 cm × 1 cm (Figure 3D). The y-axis shows the voltage change (ΔE) during the durability test, i.e., the difference between the initial voltage and the subsequent voltage value (E_i_: voltage of initial step, E_a_: voltage after durability test). During the 100 h durability tests, CCO_1:2_ and CCO_1:3_ remained constant without significant voltage changes, while CCO_1:1_ showed more noticeable voltage changes (Park Y. S. et al., 2020). As shown in Supplementary Figure 3, one reason for this result was the elemental ratios of the synthesized electrocatalysts. In the case of CCO_1:1_, there was a large amount of unreacted Cu in the spinel structure, as confirmed by ICP analysis and XPS. The led to degradation of Cu to copper hydroxide or oxide in the high potential region. Thus, CCO_1:2_ and CCO_1:3_ were found to be better electrocatalysts than CCO_1:1_ because the voltage remained constant when a constant current was applied. Combining the half-cell test results of the electrochemical catalysts, we found that both OER activity and durability were excellent when CCO was synthesized at a 1:2 ratio of Cu to Co.

**Figure 4:**
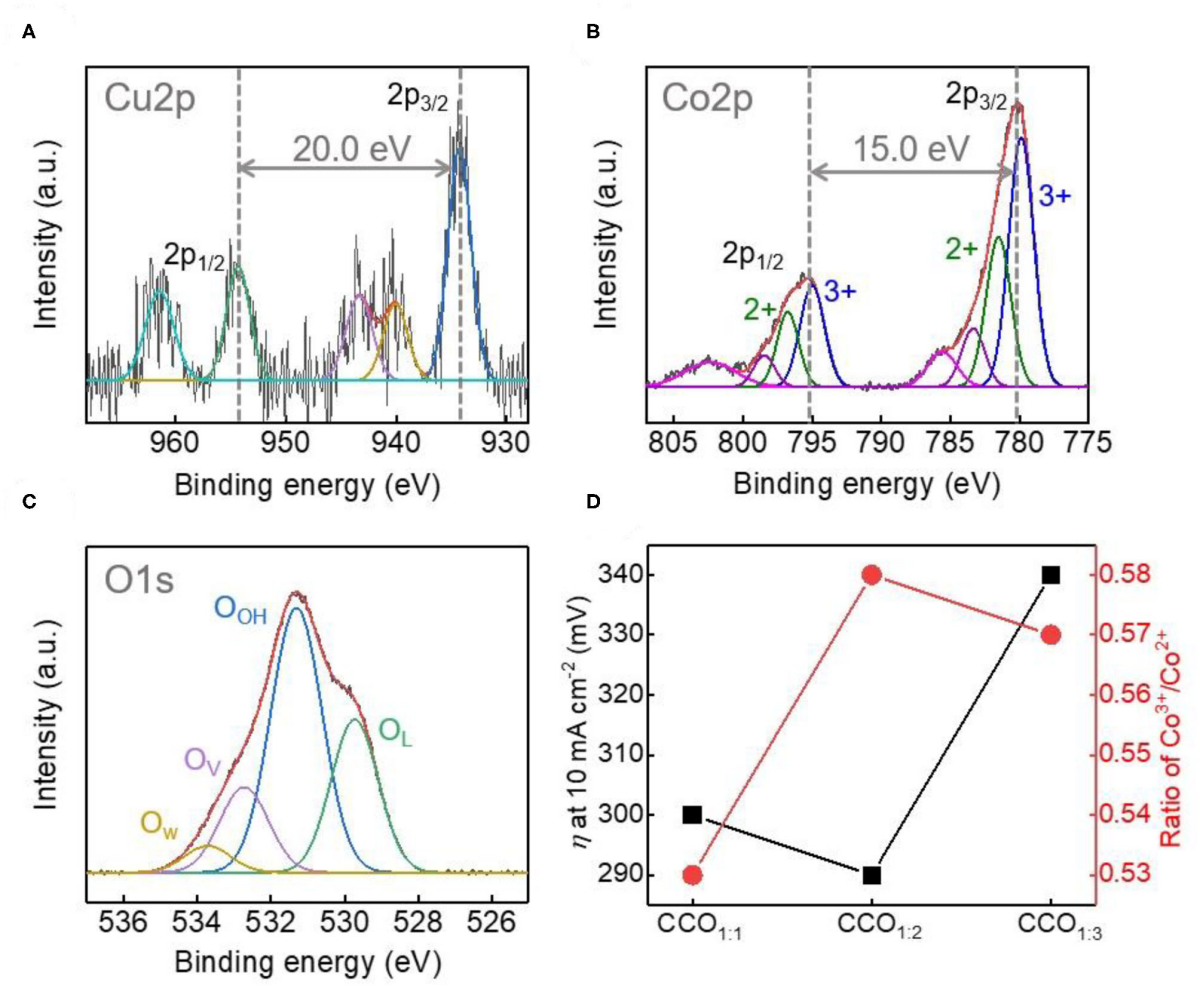
Investigation of the electronic structure of CCO_1:2_. X-ray photoelectron high resolution spectra (XPS) of Cu 2p **(A)**, Co 2p **(B)**, and O1s **(C)**. The Binding energy difference between Cu 2P_3/2_ and Cu 2p_1/2_ peak and Co 2p_3/2_ and Co 2p_1/2_ peaks are 20.0 and 15.0 eV, corresponding to that of CCO in spinel. **(D)** Comparison of the relationship between overpotential (η) at 10 mA cm^−2^ and calculated Co^3+^/Co^2+^ ratio in XPS results.

The electronic properties of Cu, Co, and O on the surfaces of the CCO electrocatalysts were analyzed *via* high resolution XPS (Figure 4 and Supplementary Figure 4). Figures 4A–C shows the XPS data for the qualitative and quantitative analysis of the electrocatalyst surface of CCO_1:2_. The Cu 2p spectrum in Figure 4A could be deconvoluted into 2p_3/2_ and 2p_1/2_ peaks at 934.3 eV, with an interval of 20.0 eV. This indicates that Cu in CCO_1:2_ was in the octahedral position and the Cu^2+^ oxidation state (Chusuei et al., 1999; De Koninck et al., 2006; La Rosa-Toro et al., 2006). As shown in Figure 4B, the Co spectrum was deconvoluted into a Co 2p_1/2_ peak and a Co 2p_3/2_ peak. The interval between these can be used to determine whether Co is in the 2+ or 3+ oxidation state (Muradov and Veziroglu, 2005), with an energy level difference of 16.0 eV indicating that Co^2+^ is dominant and 15.0 eV showing an oxidation state of Co^3+^ (Chusuei et al., 1999). In the Co 2p spectrum of CCO_1:2_, the difference between the major 2p_1/2_ and 2p_3/2_ peaks was 15.0 eV, while the deconvoluted peaks showed intervals appropriate for both Co^2+^ and Co^3+^ (Oku and Hirokawa, 1976), with peaks at 781.6 eV (Co^2+^) and 779.9 and 795.1 eV (Co^3+^). The results for both elements correspond to those reported for the spinel structure of CCO. The O 1s spectrum shown in Figure 4C was deconvoluted into four components, with O_L_ representing lattice oxygen, O_OH_ representing hydroxyls, O_V_ representing oxygen vacancies, and O_W_ representing chemisorbed water. Here, O_V_ indicates oxygen deficiency within the oxides, which a previous study has linked to improved OER response (Kent et al., 2013), as demonstrated by the synthesized CCO_1:2_.

Figure 4D shows the correlation between η and the Co^3+^/^2+^ ratios of the CCO electrocatalysts. For Co-based electrocatalysts, prior reports state that the higher the ratio of Co^3+^, the more active sites there are for oxygen evolution and thus, the better the electrocatalyst functions (Li et al., 2016). Thus, the relationship between Co^3+^ and the η value (mV, at 10 mA/cm^2^) for the different elecrrocatalysts showed that CCO_1:2_ had the highest Co^3+^/Co^2+^ value and a lower η value than those seen for electrocatalysts with ratios of 1:1 and 1:3. Therefore, it was confirmed that the excellent electrocatalytic activity of CCO_1:2_ was related to its large specific surface area and relatively high number of Co^3+^ active sites, which are favorable to the aforementioned electrochemical reactions.

**Figure 5:**
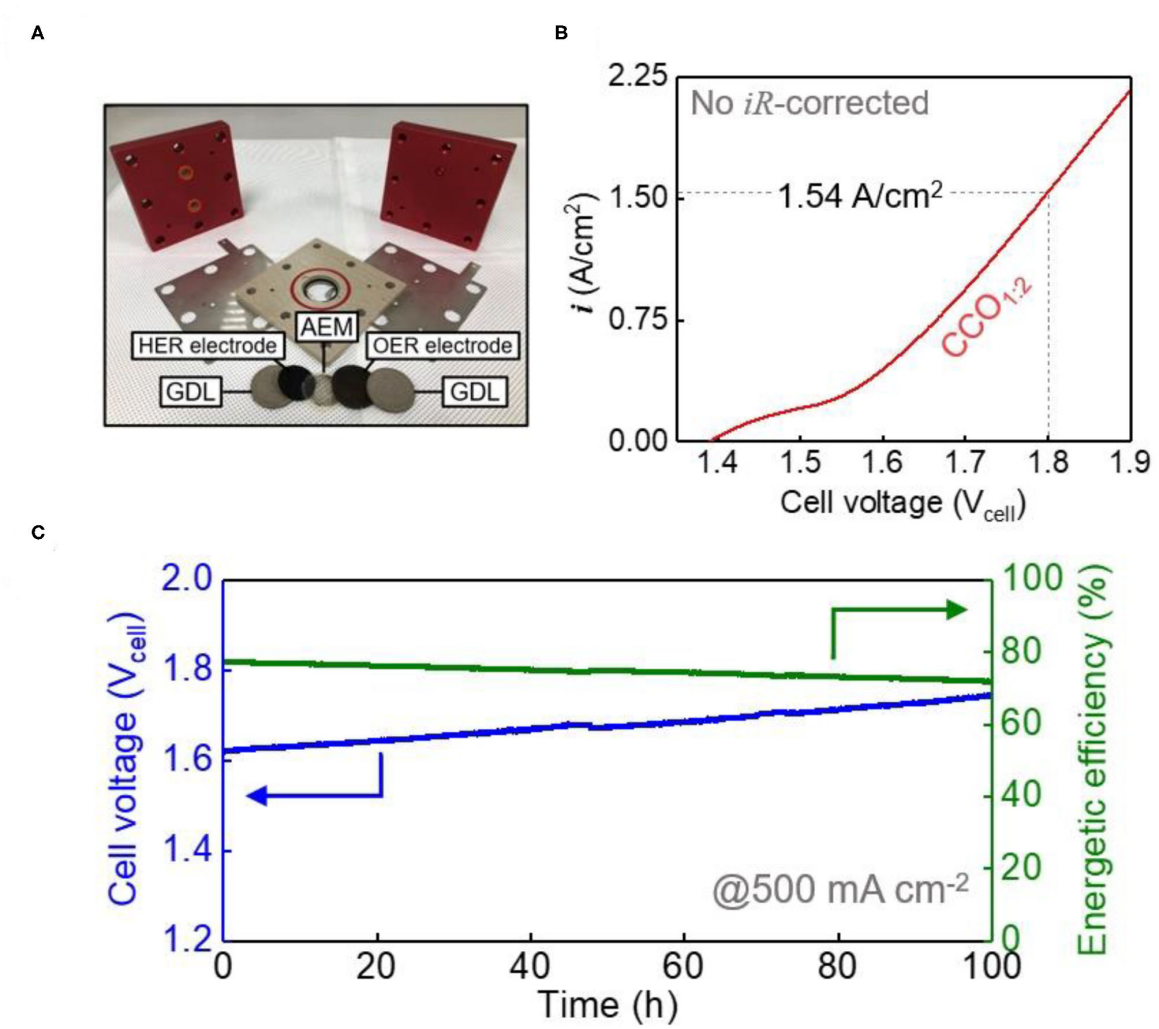
Performance of AEMEW single cell. **(A)** Photograph of components of AEMWE single cell. **(B)** The polarization curve (without iR-correction) of the AEMWE single cell with an anode of CCO_1:2_ (25 mg/cm^2^) and Pt/C (1 mg_Pt_/cm^2^) cathode electrode in 1 M KOH at 50°C. **(C)** The galvanostatic curve and energetic efficiency for the AEMWE single cell at a current density of 500 mA/cm^2^ for 100 h in 1 M KOH at 50°C.

To check the applicability of the synthesized electrocatalyst system, performance evaluation was carried out by applying it to an AEMWE single cell, configured as shown in Figure 5A. The anode was produced using a loading of 25 mg/cm^2^ of CCO_1:2_, which was the most active of the CCO ratios used in this study, while Pt/C (loading mount: 1 mg_Pt_/cm^2^) was used for the cathode (Yuan et al., 2017; Park et al., 2019). Higher operating temperatures lead to improved electrochemical reactions, but the electrolyte temperature was maintained at 60°C as higher temperatures can affect cell durability, for example by causing membrane deterioration. Figure 5B represents the LSV curve of the AEMWE single cell between 1.4 and 1.9 V_cell_, and no IR compensation was made for performance verification under actual operating conditions. A value of 1.54 A/cm^2^ was obtained at 1.8 V_cell_, which shows excellent performance compared to recent papers on the application of non-precious metal electrocatalysts to anodes (Supplementary Table 1). Although synthesized electrocatalysts perform well in half-cell units, it is not easy to show the same performance when systems such as single cell are used (Xu et al., 2019). Moreover, long-term durability at the high current density required to meet the standards of industrial hydrogen production (400–500 mA/cm^2^) is very important. Figure 5C shows the stabilities of the AEMWE single cell when CCO_1:2_ was used. The durability experiments at high current density were conducted at 500 mA/cm^2^ per unit area, and showed only a 7% increase in voltage over 100 h compared to the initial performance, indicating good durability. Calculating the energy efficiency using Equation (5) showed 71% efficiency even after the 100 h durability test. Therefore, CCO_1:2_ electrocatalyst anodes were found to be applicable to commercially available AEMWEs.

(5) $$\begin{array}{l} \text{Energetic efficiency}=\frac{E_{Output}}{E_{Input}}=\frac{V_{H2}\cdot H_{O}}{W_{h}}\;\times 100 \end{array}$$

Where W_h_ is the electric power required to produce hydrogen, H_0_ is the calorific value of hydrogen (10.8 × 106 J/m^3^, lower heating value), and V_H2_ is the volume of hydrogen gas.

## Conclusion

In this study, the high performance non-precious metal-based oxygen evolution electrocatalyst CCO was synthesized through a coprecipitation method and thermal oxidation treatment, and the changes in the electrocatalytic properties were examined as the ratio of Cu to Co was adjusted. Going beyond this synthesis of a highly active electrocatalyst, CCO was also used as an anode for an AEMWE single cell to examine whether the developed electrocatalyst could perform in an actual AEMWE system. Of the ratios compared, the CCO_1:2_ electrocatalyst with a Cu to Co ratio of 1:2 showed the best activity and durability, most likely due to the effect of a high ratio of Co^3+^ that gives a high specific surface area and abundant active sites for oxygen evolution. The single cell evaluation of the CCO_1:2_ electrocatalyst also showed a high activity of 1.54 A/cm^2^ at 1.8 V_cell_, while the durability evaluation at a high current density (500 mA/cm^2^) showed an energy efficiency of 71% after 100 h.

## Data Availability Statement

All datasets presented in this study are included in the article/Supplementary Material.

## Author Contributions

C-YK, J-YJ, and JY synthesized the electrocatalysts and evaluated their electrochemical properties. C-YK and JY performed the physical characterizations. J-YJ and JY fabricated MEA for AEMWE. YP and JJ tested AEMWE single cell's performance. HP measured BET surface area and pore distribution for materials. YK and SC coordinated and supervised the overall project. All authors reviewed the final manuscript.

## Conflict of Interest

The authors declare that the research was conducted in the absence of any commercial or financial relationships that could be construed as a potential conflict of interest.

The handling editor declared a past co-authorship with several of the authors SC, JJ, JY, YP, and YK.
