# Supplementary material for: Effect of Copper Cobalt Oxide Composition on Oxygen Evolution Electrocatalysts for Anion Exchange Membrane Water Electrolysis
Source: Front Chem. 2020 Nov 4;8:600908. doi: 10.3389/fchem.2020.600908 (PMC7741587; doi:10.3389/fchem.2020.600908)
Supplement: Supplementary file 1 [file Data_Sheet_1.docx]

**Electronic Supplementary Information (ESI) for**

**Effect of copper cobalt oxide composition on oxygen evolution electrocatalysts for anion exchange membrane water electrolysis**

Chae-Yeon Kwon^a,b,†^, Jae-Yeop Jeong^a,c,†^, Juchan Yang^a,†^, Yoo Sei Park^a,c^, Jaehoon Jeong^a^, Honghyun Park^d^, Yangdo Kim^c,*^, Kyoung-Seok Moon^b,*^, Sung Mook Choi^a,*^

a Materials Center for Energy Convergence, Surface Technology Division, Korea Institute of Materials Science (KIMS), Changwon, 51508, Republic of Korea

b School of Materials Science and Engineering, Gyeongsang National University, Jinju, 52828, Republic of Korea

c Department of Materials Science and Engineering, Pusan National University, Busan, 46241, Republic of Korea

d Department of Advanced Biomaterials Research, Materials Processing Innovation Research Division, Korea Institute of Materials Science (KIMS), Changwon, 51508, Republic of Korea

† These authors contributed equally to this work.

Email addresses: yangdo@pusan.ac.kr (Prof. Y. Kim), ksky.moon@gnu.ac.kr (Prof. K. S. Moon), akyzaky@kims.re.kr (Dr. S. M. Choi)


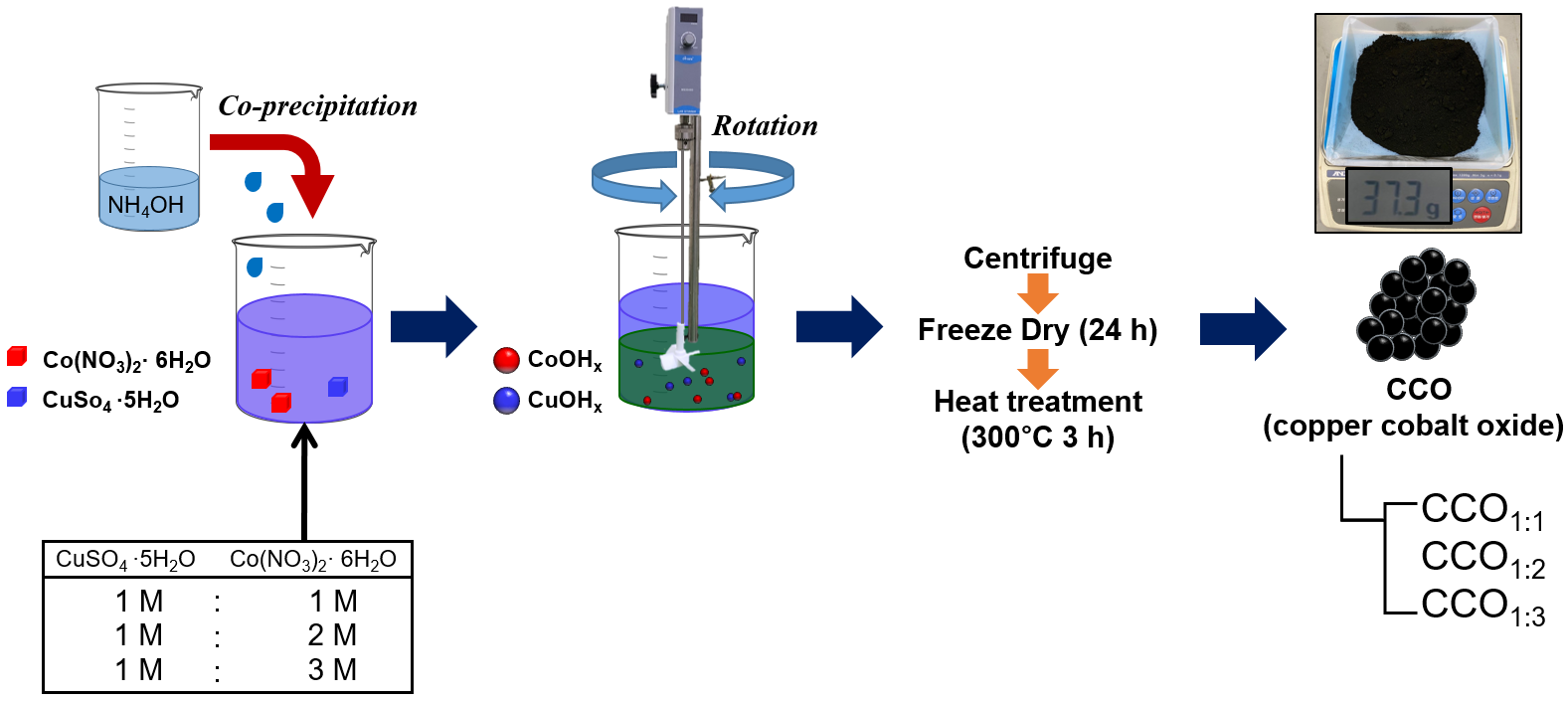


**Fig. S1. The synthesis process of CCO electrocatalyst according to the change of precursor ratio.**


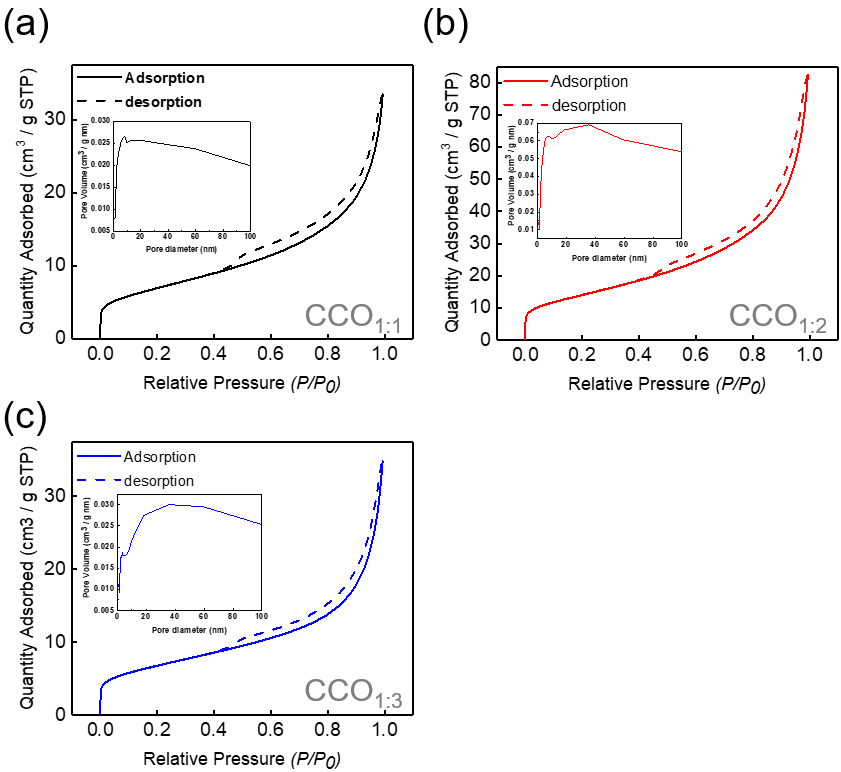


**Fig. S2. Nitrogen adsorption/desorption isotherm and pore size distribution (inset) at 77 K.** (a) CCO_1:1_. (b) CCO_1:2_. (c) CCO_1:3_.


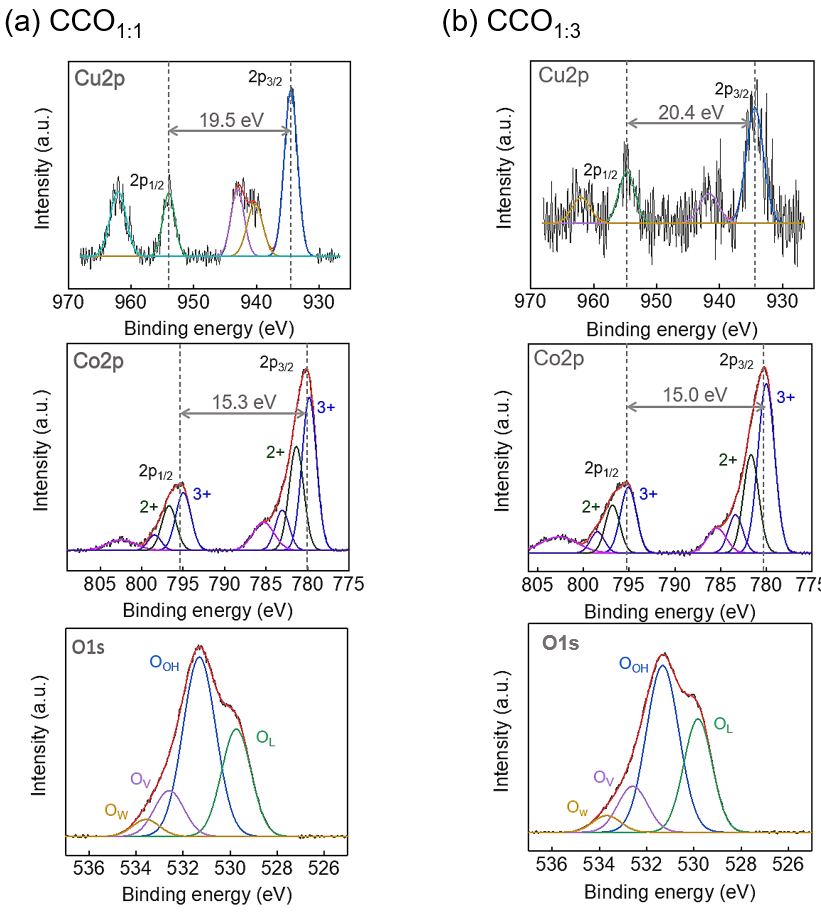


**Fig. S3. (a and b) X-ray photoelectron spectra of Cu 2p, Co2p and O1s of CCO_1:1_ and CCO_1:3_.** Gaussian-Lorentzian distribution was used for curve fitting after Shirley background correction. The two main peaks at binding energies of Co2P_3/2_ and Co2P_1/2_ could be deconvoluted into four peak components. The separated binding energy gap between the main peaks in Co2P is 15.0 ~15.3 eV. The binding energy difference between the Cu2P_1/2_ and CuP_3/2_ peak is around 20 eV, and the deconvoluted peaks could be best fitted to the spinel oxides. Both results are consistent with that of copper cobalt oxide (CCO) spinel structure.


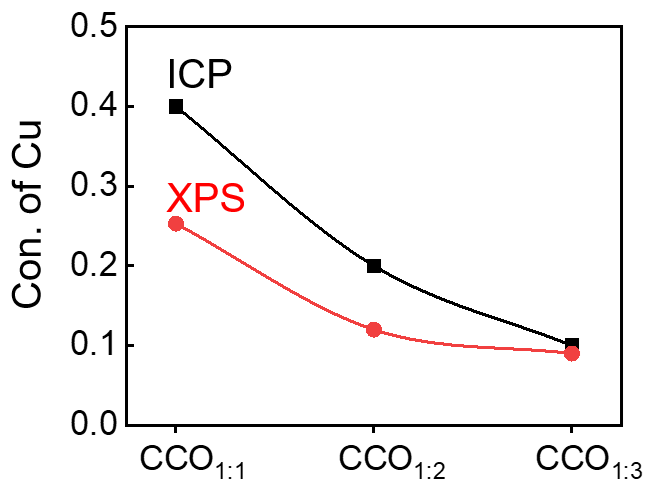


**Fig. S4. The change of concentration of Cu in CCO electrocatalyst with a different precursor ratio. The result of both ICP and XPS show the same tendency**

**Table S1.** **Comparison of the AEMWE single cell reported performance with non-precious OER electrocatalyst at 1.8 V_cell_.**


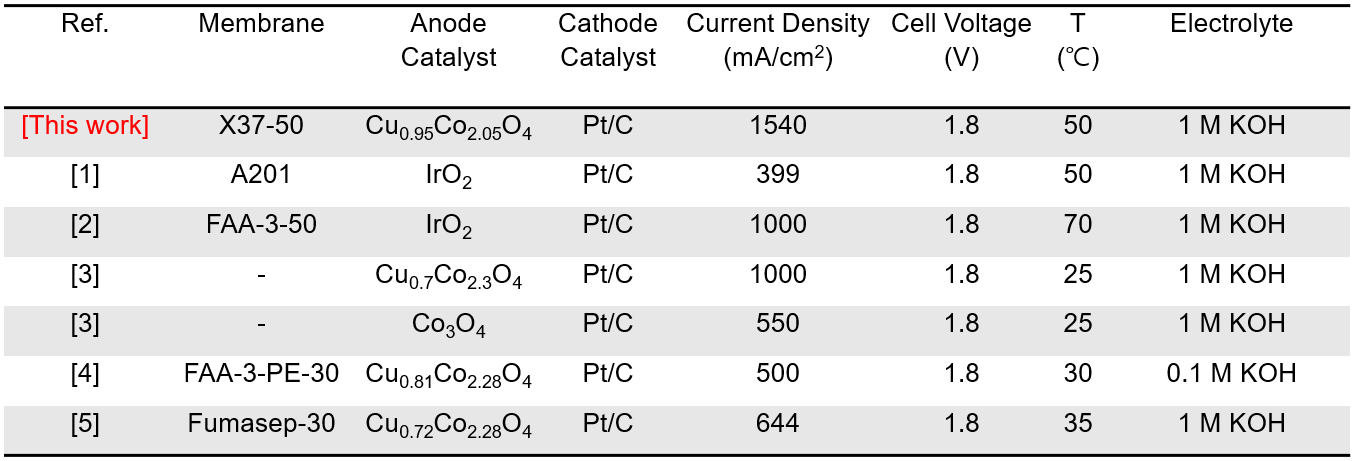


[1] Y. Leng, G. Chen, A.J. Mendoza, T.B. Tighe, M.A. Hickner, and C.-Y. Wang. (2012). ‘Solid-state water electrolysis with an alkaline membrane’. *J. Am. Chem. Soc.* 134, 9054-9057. doi:[10.1021/ja302439z](https://pubs.acs.org/doi/abs/10.1021/ja302439z)

[2] J.E. Park, S.Y. Kang, S.-H. Oh, J.K. Kim, M.S. Lim, C.-Y. Ahn, Y.-H. Cho, and Y.-E. Sung. (2019). ‘High-performance anion-exchange membrane water electrolysis’. *Electrochim. Acta*. 295, 99-106. dio:[10.1016/j.electacta.2018.10.143](https://doi.org/10.1016/j.electacta.2018.10.143)

[3] X. Wu, and K. Scott. (2011). ‘Cu_x_Co_3−x_O_4_ (0 ≤ x < 1) nanoparticles for oxygen evolution in high performance alkaline exchange membrane water electrolysers’. *J. Mater. Chem.* 21, 12344-12351. doi:[10.1039/C1JM11312G](https://doi.org/10.1039/C1JM11312G)

[4] W.-S. Choi, M.J. Jang, Y.S. Park, K.H. Lee, J.Y. Lee, M.-H. Seo, and S.M. Choi. (2018). ‘Three-dimensional honeycomb-like Cu_0.81_Co_2.19_O_4_ nanosheet arrays supported by Ni foam and their high efficiency as oxygen evolution electrodes’. *ACS Appl. Mater. Interfaces*. 10, 38663-38668. dio:[10.1021/acsami.8b12478](https://pubs.acs.org/doi/10.1021/acsami.8b12478)

[5] S.M. Park, M.J. Jang, Y.S. Park, J. Lee, J.-y. Jeong, J. Jung, M.-K. Choi, Y.-S. Noh, M.-H. Seo, H.J. Kim, J. Yang, Y.D. Kim, and S.M. Choi. (2020). ‘Synthesis and characterization of the Cu_0.72_Co_2.28_O_4_ catalyst for oxygen evolution reaction in an anion exchange membrane water electrolyzer’. *Korean J. Met. Mater.* 58, 49-58. dio:[10.3365/KJMM.2020.58.1.49](http://dx.doi.org/10.3365/KJMM.2020.58.1.49)
